# Supplementary material for: β-sheet stabilization of the island domain underlies ligand-induced LRR-RP activation of plant immune signaling
Source: Nat Commun. 2025 Dec 8;16:10958. doi: 10.1038/s41467-025-66119-7 (PMC12686451; doi:10.1038/s41467-025-66119-7)
Supplement: Supplementary file 1 — Supplementary Information [file 41467_2025_66119_MOESM1_ESM.pdf]

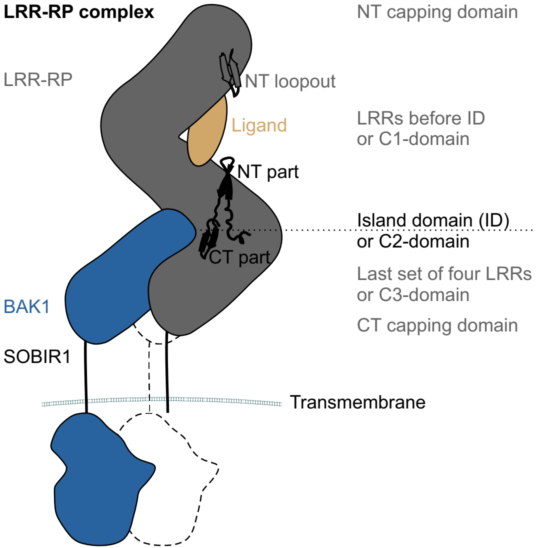

Supplementary Fig. 1: Graphical representation of the LRR-RP – ligand – BAK1 – SOBIR1 receptor complex. Overview of the LRR-RP nomenclature.

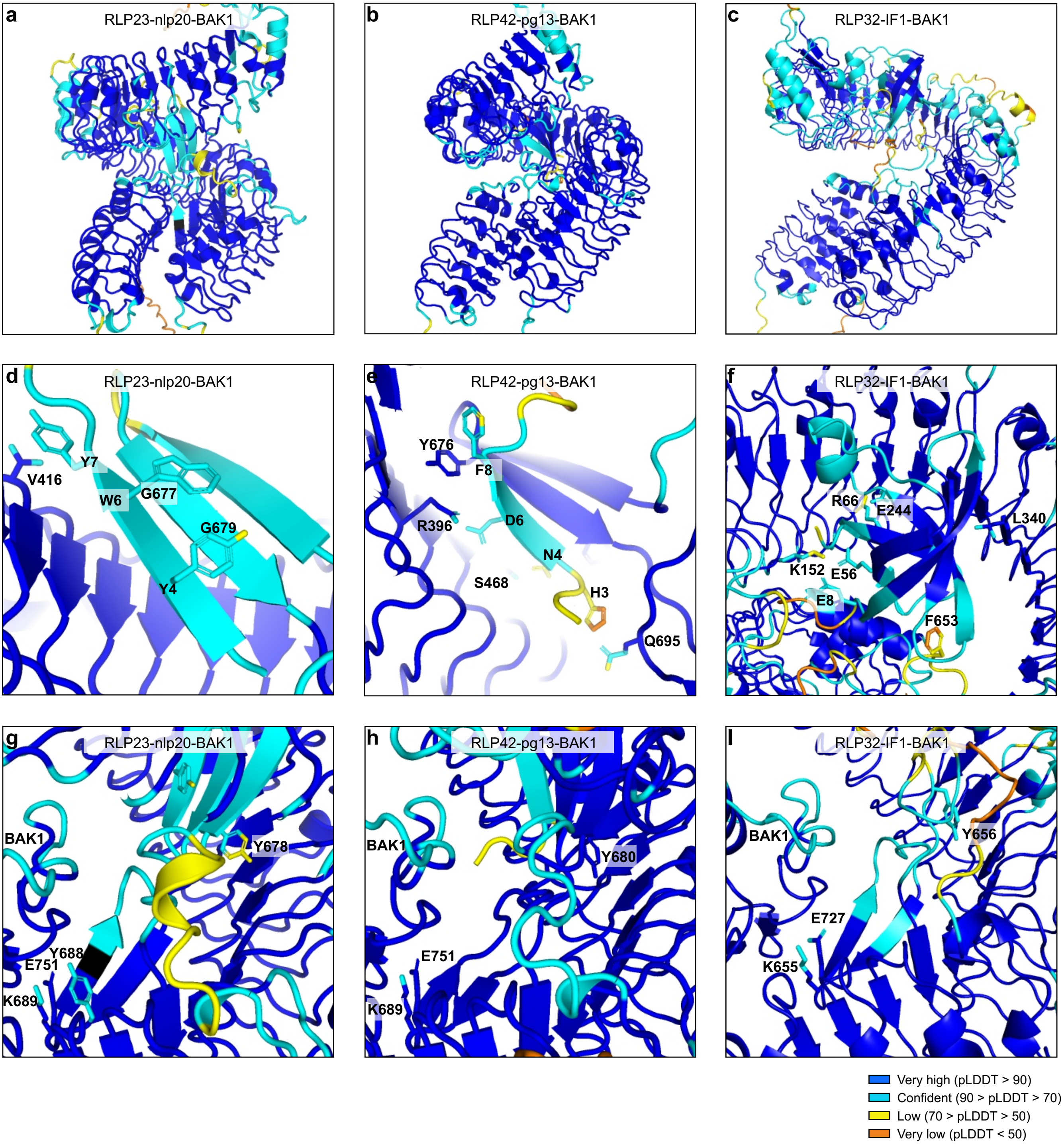

Supplementary Fig. 2: Visualization of the predicted local distance difference test (pLDDT) score for the AF3 predictions of RLP23 (left), RLP42 (middle) and RLP32 (right).
pLDDT is a per-atom confidence estimate on a 0-100 scale where a higher value indicates higher confidence and usually a more accurate prediction, here depicted for the LRR-RP complex predictions using the pymol extension pymol-color-alphafold, coloring as per legend. **a-c)** Overview of the complete receptor complex prediction. **d-f)** Zoom in on the ligand-binding interface. **g-i)** Zoom in on the interaction interface between the NT part of the ID and BAK1. Pdb files can be found in Supplementary Data 1.

a

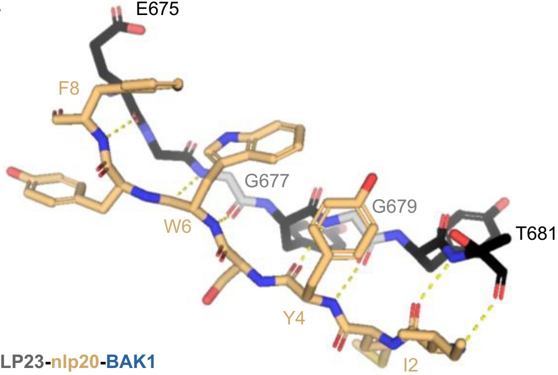

RLP23-nlp20-BAK1

RLP23	a.a	atom	nlp20	a.a	atom	distance (Å)
THR	681	O	ILE	2	N	3.06
THR	681	N	ILE	2	O	2.91
GLY	679	O	TYR	4	N	2.98
GLY	679	N	TYR	4	O	2.86
GLY	677	O	TRP	6	N	3.02
GLY	677	N	TRP	6	O	3.06
GLU	675	O	PHE	8	N	2.85

b

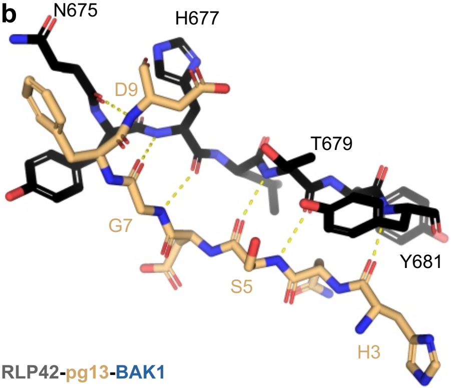

RLP42-pg13-BAK1

RLP42	a.a	atom	pg13	a.a	atom	distance (Å)
TYR	681	N	HIS	3	O	2.8
THR	679	O	SER	5	N	2.96
THR	679	N	SER	5	O	2.9
HIS	677	O	GLY	7	N	3.06
HIS	677	N	GLY	7	O	2.89
ASN	675	O	ASP	9	N	2.94

Supplementary Fig. 3: Visualization of the predicted antiparallel beta-sheet interactions of RLP23 (a) and RLP42 (b) with their respective ligands nlp20 and pg13. The analysis was performed using PDBePISA and the respective tripartite receptor complex predictions. H-bonds are indicated and the correlating predicted distances are depicted within the respective tables.

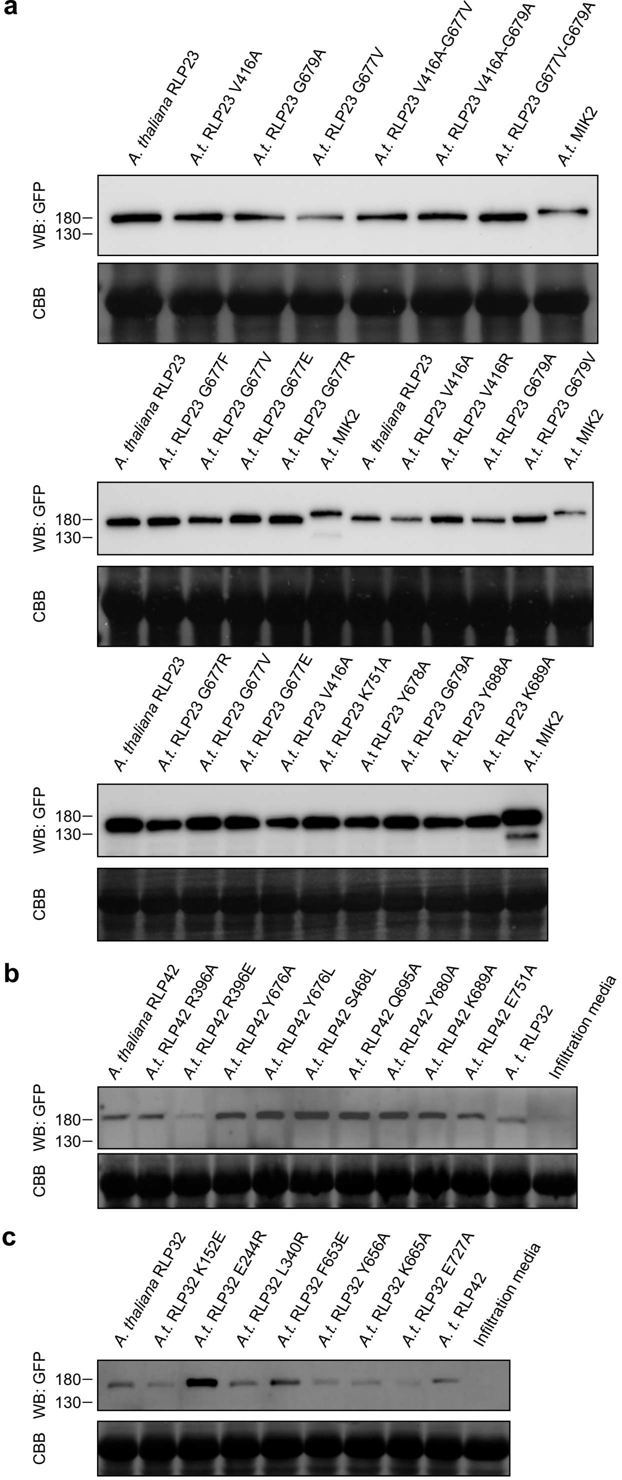

Supplementary Fig. 4: Western blotting following heterologous expression of the LRR-RPs and their respective variants in *N. benthamiana*.

a-c) Western blot 72 h post-Agrobacterium infiltration of respectively RLP23, RLP42 and RLP32. The western blots were probed with α-GFP (B-2) HRP as the receptor had a C-terminal GFP tag (top) and subsequently stained with CBB as a loading control (bottom). MIK2, RLP32 and RLP42 were used as positive control for heterologous expression respectively in a, b and c, as they share the same expression vector as the receptor (variants) of interest.

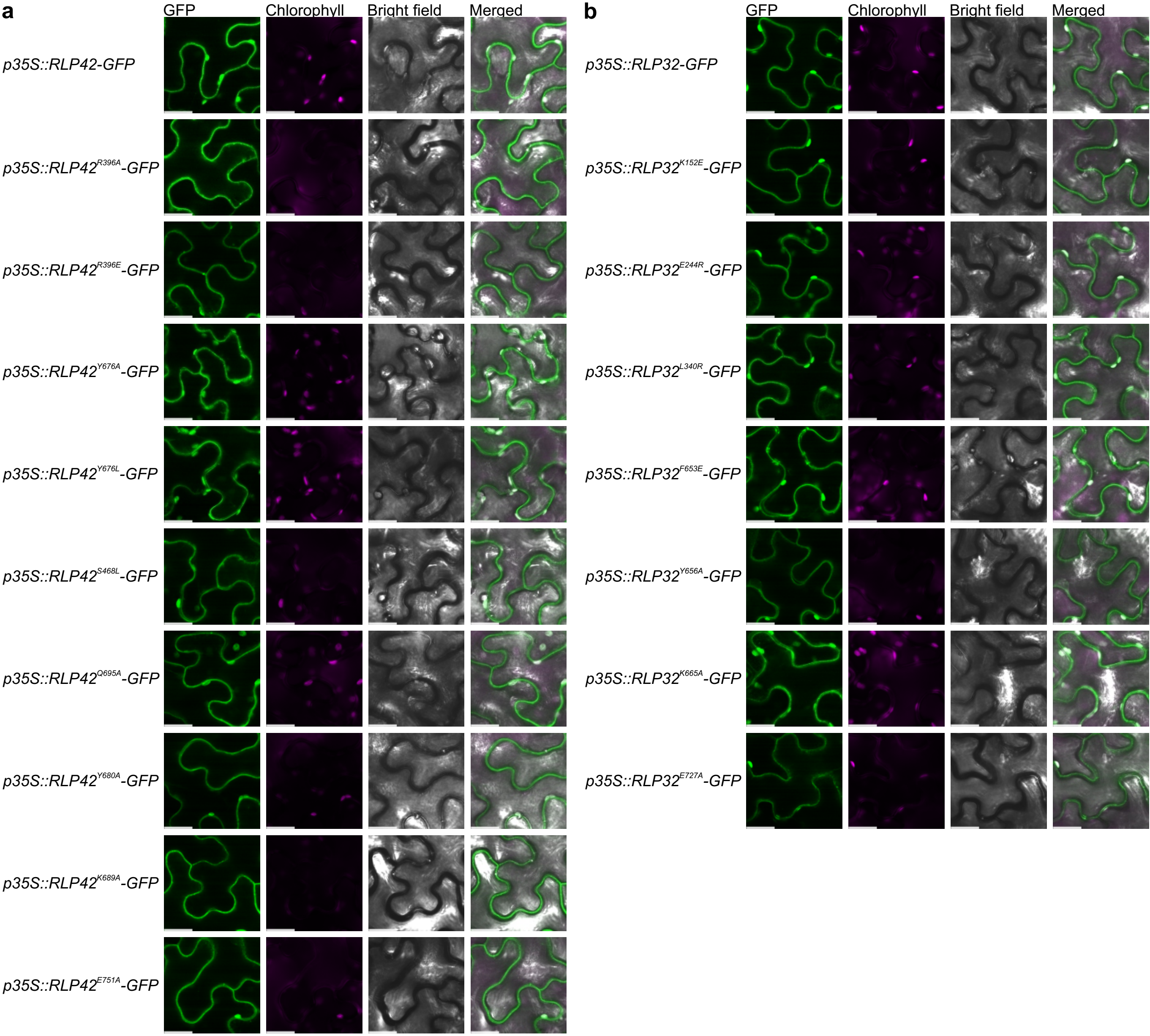

Supplementary Fig. 5: Confocal microscopy following heterologous expression of the LRR-RPs and their respective variants in *N. benthamiana*.

a-b) Confocal microscopy (GFP, Chlorophyll B and Bright Field) following Agrobacterium infiltration of respectively RLP42 (a) and RLP32 (b) and their respective variants (72 h). All confocal microscopy images were taken with the same image settings and identically modified. White scale bar represents 20 µm.

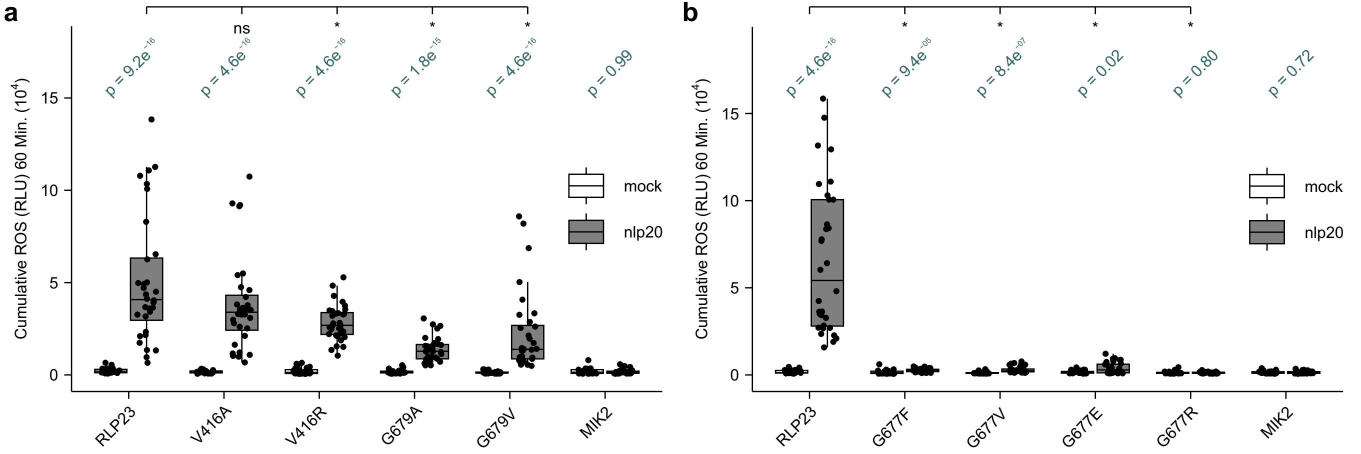

Supplementary Fig. 6: Single AA changes to diverse residues within the predicted ligand-binding interface affect the functionality of the RLP23. a-b) ROS production (4 to 60 min) in cumulative RLUs post treatment with H_2O (white) or 1 µM nlp20 (gray). Eight independent biological replicates (n = 8 plants) were performed, with each biological replicate represented by at least three technical replicates. Box plots indicate the median and the interquartile range (IQR), the whiskers extend to the most extreme data points within 1.5 times the IQR. Significance was tested by performing non-parametric two-sided Wilcoxon-Mann-Whitney tests between both mock and ligand RLP23 (variants) (depicted in green), as well as ligand-treated RLP23 vs specific variants, without adjustments for multiple comparisons. The asterisks indicate a significant difference of $p < 0.05$.

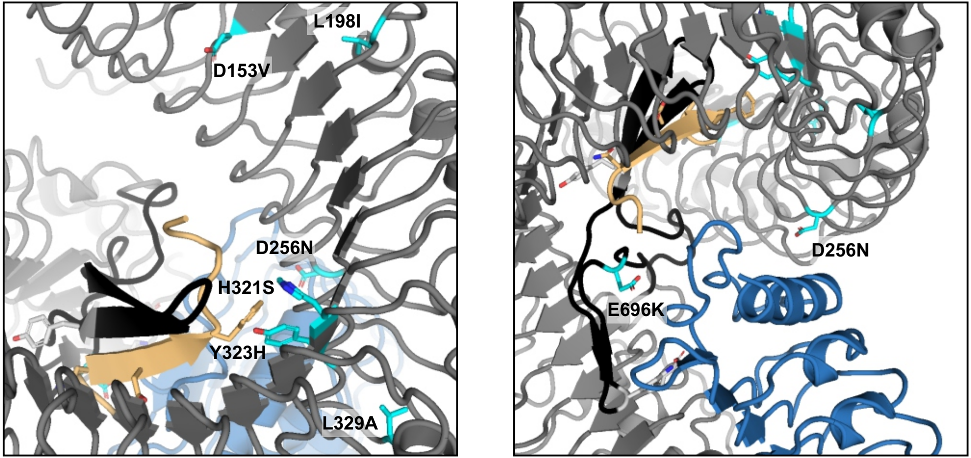

Supplementary Fig. 7: Earlier characterized single AA changes affect ligand binding and BAK1 recruitment by RLP42. Structural representations of the tripartite complexes of RLP42-pg13-BAK1. The ID is highlighted in black, other LRR-RP domains in dark grey. The ligand is depicted in yellow, and BAK1 in blue. Residues highlighted in light blue were earlier shown to affect RLP42 functionality [9]. L198 and L329 are predicted to be part of the hydrophobic core of the RLP42 LRR domain. Hence, mutations on those residues might affect core packing of the LRR domain in turn potentially abolishing ligand binding and/or BAK1 recruitment. H321S and Y323H substantially reduced ligand-binding and BAK1 recruitment and are predicted to reside at the surface of the LRR domain in proximity to the interaction interface between the ligand and the ID. Similarly, E696K affected ligand binding and BAK1 recruitment and is localized within the RLP42 ID. Finally, the D256N mutation results in the N-x-T motif, which is a consensus sequence for N-glycosylation 27. Consequently, glycosylation in the inner surface of the LRR domain likely provides steric hindrance altering ligand binding and BAK1 recruitment upon ligand binding. Pdb files can be found in Supplementary Data 1.

Supplementary Table

Table S1: **Primers used in this study.**

Gene	Varian	Orientation	Sequence
AT2G3268	G677R	fw	CGAAGGCCGCTATGGTTATACAGATGC
AT2G3268	G677R	rev	CATAGCGGCCTTCGTCGAACAG
AT2G3268	G677E	fw	CGAAGGCGAGTATGGTTATACAGATGCTT
AT2G3268	G677E	rev	TAACCATACTCGCCTTCGTCGAACA
AT2G3268	G677V	fw	CGAAGGCGTCTATGGTTATACAGATGCTTTAGATTT
AT2G3268	G677V	rev	ACCATAGACGCCTTCGTCGAACA
AT2G3268	G677F	fw	CGAAGGCTTCTATGGTTATACAGATGCT
AT2G3268	G677F	rev	CCATAGAAGCCTTCGTCGAACAGC
AT2G3268	G679V	fw	GCTATGTTTATACAGATGCTTTAGATTTGC
AT2G3268	G679V	rev	CTGTATAAACATAGCCGCCTTCG
AT2G3268	G679A	fw	GGCTATGCTTATACAGATGCTTTAGAT
AT2G3268	G679A	rev	CTGTATAAGCATAGCCGCCTTC
AT2G3268	Y678A	fw	GCGGCGCTGGTTATACAGATGCTTTA
AT2G3268	Y678A	rev	TAACCAGCGCCGCCTTCGTC
AT2G3268	V416R	fw	GAGTTGCGGTATATAGACATATCCAA
AT2G3268	V416R	rev	CTATATACCGCAACTCCTTGAGG
AT2G3268	V416A	fw	AGTTGGCGTATATAGACATATCCAAC
AT2G3268	V416A	rev	CTATATACGCCAACTCCTTGAGG
AT2G3268	Y688A	fw	TTGCAAGCCAAAGGTCTACACATGGA
AT2G3268	Y688A	rev	ACCTTTGGCTTGCAAATCTAAAGCATCTGTATAACC
AT2G3268	K689A	fw	GCAATACGCAGGTCTACACATGGAGC
AT2G3268	K689A	rev	TAGACCTGCGTATTGCAAATCTAAAGCATCTGTATAACC
AT2G3268	E751A	fw	GAATCTCGCGTCACTAGACATGTCAAGA
AT2G3268	E751A	rev	CTAGTGACGCGAGATTCTCAAGATTGGC
AT2G3268	G677V	fw	CGAAGGCGTCTATGCTTATACAGATGCTTTAGATTT
AT2G3268	G677V	rev	AGCATAGACGCCTTCGTCGAACA
AT3G0565	WT	fw	CTATTCTAGTCGACCTGCAGGCGGCCGCACTAGTATGAAAGACTCTTGGAACTCAAC
AT3G0565	WT	rev	GTGAACAGCTCCTCGCCCTTGCTCACCATACTAGTTTGCTTTCTCCTCAATCTTTTTTC
AT3G0565	K152E	fw	CTCATCTTACCACTCTCGACCTTTCTGAAAACTATTTCAGTGGTGGGATTCCTTC
AT3G0565	K152E	rev	GAAGGAATCCCACCACTGAAATAGTTTTCAGAAAGGTCGAGAGTGGTAAGATGAG
AT3G0565	E244R	fw	GAGCTCACTCTCTAACTTGGAGTACTTTCGGGCATGGGGCAACGCTTTTACTGGAAC
AT3G0565	E244R	rev	GTTCCAGTAAAAGCGTTGCCCCATGCCCGAAAGTACTCCAAGTTAGAGAGTGAGCTC
AT3G0565	L340R	fw	GTATCTTCACGAATCTCAAGTCGCTCCAACGTCTTAACCTATCTCATTTGAACACCAC
AT3G0565	L340R	rev	GTGGTGTTCAAATGAGATAGGTTAAGACGTTGGAGCGACTTGAGATTCGTGAAGATAC
AT3G0565	F653E	fw	CTCAAGAGAAATACATGGGAGACTCTGAACGATATTACCATGATTCAGTGGTTTTG
AT3G0565	F653E	rev	CAAAACCACTGAATCATGGTAATATCGTTCAGAGTCTCCCATGTATTTCTCTTGAG
AT3G0565	Y656A	fw	GAAATACATGGGAGACTCTTTTCGATATGCCCATGATTCAGTGGTTTTGATGAATAAAG
AT3G0565	Y656A	rev	CTTTATTCATCAAAACCACTGAATCATGGGCATATCGAAAAGAGTCTCCCATGTATTTC
AT3G0565	K665A	fw	TACCATGATTCAGTGGTTTTGATGAATGCAGGCTTAGAGATGGAGCTGGTACGCATC
AT3G0565	K665A	rev	GATGCGTACCAGCTCCATCTCTAAGCCTGCATTCATCAAAACCACTGAATCATGGTA
AT3G0565	E727A	fw	CATCTATGGGGAATCTTAGAGAGCTCGCATCACTGGACGTTTCTCAAAACAAGCTTTC
AT3G0565	E727A	rev	GAAAGCTTGTTTTGAGAAACGTCCAGTGATGCGAGCTCTCTAAGATTCCCCATAGATG
