# Supplementary material for: β-sheet stabilization of the island domain underlies ligand-induced LRR-RP activation of plant immune signaling
Source: Nat Commun. 2025 Dec 8;16:10958. doi: 10.1038/s41467-025-66119-7 (PMC12686451; doi:10.1038/s41467-025-66119-7)
Supplement: Supplementary file 2 — Description of Additional Supplementary Information [file 41467_2025_66119_MOESM2_ESM.pdf]

Description of Additional Supplementary Files

File Name: Supplementary Data 1
Description: AF3 predicted structures (.pdb).

File Name: Supplementary Data 2
Description: Plasmid maps of constructs used in this study.
